# Safety and efficacy of second-generation all-suture anchors in labral tear arthroscopic repairs: prospective, multicenter, 1-year follow-up study

**DOI:** 10.1016/j.jseint.2024.04.008

**Published:** 2024-04-27

**Authors:** Andrew L. Wallace, Emilio Calvo, Jordi Ardèvol Cuesta, Riccardo Lanzetti, Gonzalo Luengo-Alonso, Andrew S. Rokito, Edwin E. Spencer, Marco Spoliti

**Affiliations:** a Fortius Clinic, London, UK; b Hospital Universitario Fundación Jiménez Díaz, Madrid, Spain; c Asepeyo Hospital Sant Cugat, Barcelona, Spain; d Azienda Ospedaliera San Camillo Forlanini, Rome, Italy; e NYU Langone Orthopedic Hospital, New York, NY, USA; f OrthoTennessee, Knoxville, TN, USA

**Keywords:** Labral tear, Shoulder instability, Glenoid labrum, All-soft suture anchor, Shoulder repair, Arthroscopic repair

## Abstract

**Background:**

This study’s primary aim was to assess the safety and performance of second-generation all-soft suture anchors following arthroscopic labral tear repair.

**Methods:**

This prospective, multicenter study was conducted by 6 surgeons at 6 sites in Europe and the United States between November 2018 and August 2020. Patients who required shoulder arthroscopic repair, for a range of labral injuries, were treated with a second-generation all-soft suture anchor. The primary outcome was clinical success rate (percentage of patients without signs of failure and/or reintervention) at 6 months. Secondary outcomes included clinical success rate at 12 months, intraoperative anchor deployment success rate, and patient-reported outcomes (PROs) at 6 and 12 months, including visual analog scale (VAS) pain assessment, VAS satisfaction assessment, EQ-5D-5L Index Score, EQ-5D-5L VAS Health Score, Rowe Shoulder Score for Instability, American Shoulder and Elbow Surgeons score, and Constant-Murley Shoulder Score. Serious adverse events and serious adverse device effects were collected throughout the study.

**Results:**

Forty-one patients were enrolled (mean age, 28.2 years; 87.8% male, 12.2% female). Clinical success was achieved in 27/28 and 31/32 patients at 6 months and 12 months, respectively. Anchor deployment had a 100% success rate. Significant improvements over baseline were reported for all PROs except Constant-Murley Shoulder (6 months) and VAS Satisfaction Score (12 months). One patient experienced 1 serious adverse event and 1 patient experienced 1 serious adverse device effect.

**Conclusion:**

Second-generation all-soft suture anchors used in this study demonstrated a high clinical success rate, a favorable safety profile, and patients exhibited significant improvement in PROs.

Suture anchors, used in the operative repair of labral tears to affix glenoid labrum to the bone, continue to evolve. Traditionally, these anchors have been made of metal, nonbiodegradable, polymer materials, or biodegradable materials.^25^ Each of these materials is associated with potential safety concerns. Metal anchors can become prominent and cause chondral damage, can be difficult to revise or reposition, and can distort and render magnetic resonance imaging of the joint unusable.^12^ Plastic anchors can also become prominent, brittle, loose, and can toggle, break in the joint, or migrate. Biodegradable anchors can break on insertion, as well as lead to osteolysis, loosening, failure of labral healing, and cause concerns about liberation products of degradation on cartilage.^5^

All-soft suture anchors offer several advantages over solid suture anchors, including reduced bone loss and limited joint damage in case of anchor failure.^10^ The small diameter of these anchors may also be advantageous as it enables fixation at multiple points, which is known to reduce the risk of recurrent instability. A small tunnel diameter may also minimize the likelihood of glenoid rim fracture.^4^ However, when compared with traditional solid suture anchors, first-generation all-soft suture anchors required less force for 2-mm displacement, indicating potential micromotion, which, in turn, could lead to disrupted labrum healing as well as bone damage and resorption.^10^

Compared with first-generation all-soft suture anchors, second-generation all-soft suture anchors used in this study have been shown to be superior in terms of displacement after cyclic loading and pull-out strength.^7^ This performance advantage may be attributed to an active deployment mechanism that does not require manual tensioning via the pulling motion. There is also greater convenience for the surgeon, as these anchors are designed for single use and do not require additional instrument trays or devices for deployment.

This study is the first prospective series evaluating a second-generation all-soft suture anchor for arthroscopic repair of labral tears and reporting a comprehensive range of patient-reported outcomes (PROs). This study’s primary aim was to assess the safety and performance of second-generation all-soft suture anchors following arthroscopic labral tear repair. We hypothesized that these anchors would have a high clinical success rate and a favorable safety profile in patients undergoing arthroscopic repair of shoulder labral tears.

## Materials and methods

### Study design

This prospective, multicenter study was conducted by 6 surgeons at 6 sites in Europe and the United States between November 2018 and August 2020. Patients who met the following criteria were included in the study: age ≥ 18 years at the time of surgery; shoulder labral indications to undergo arthroscopic repair with the study device that were confirmed by physical findings, patient symptoms, and radiographic findings; sufficient bone quality to allow proper placement of the anchor; and willingness and ability to make all required study visits and follow directions.

Conversely, patients were excluded if they participated in the treatment period of another clinical trial within 30 days of visit 1 (preoperative); were women who were pregnant, nursing, or of child-bearing potential who were not using highly effective birth control measures; met the definition of a “vulnerable subject” per ISO 14155:2011; had a history of poor compliance with medical treatment; had contraindications or hypersensitivity to the use of the study device, or their components (eg, silicone, polyester [where material sensitivity was suspected, appropriate tests were performed, and sensitivity ruled out prior to implantation]); had prior ipsilateral surgeries performed on the joint space; had pathological conditions of the soft tissues or bone, or comminuted bone surface, which would compromise secure anchor or suture fixation; had physical conditions which would eliminate, or tend to eliminate, adequate anchor support and impair healing; had a history of epilepsy; had glenoid and/or humeral bone loss considered excessive by the treating orthopedic surgeon; and had multidirectional instability or psychosomatic voluntary shoulder subluxation.

This study was performed in compliance with the ethical principles of the World Medical Association Declaration of Helsinki, and institutional review board approval was obtained for each investigational site. All patients provided voluntary informed consent in writing before enrollment.

**Figure 1:**
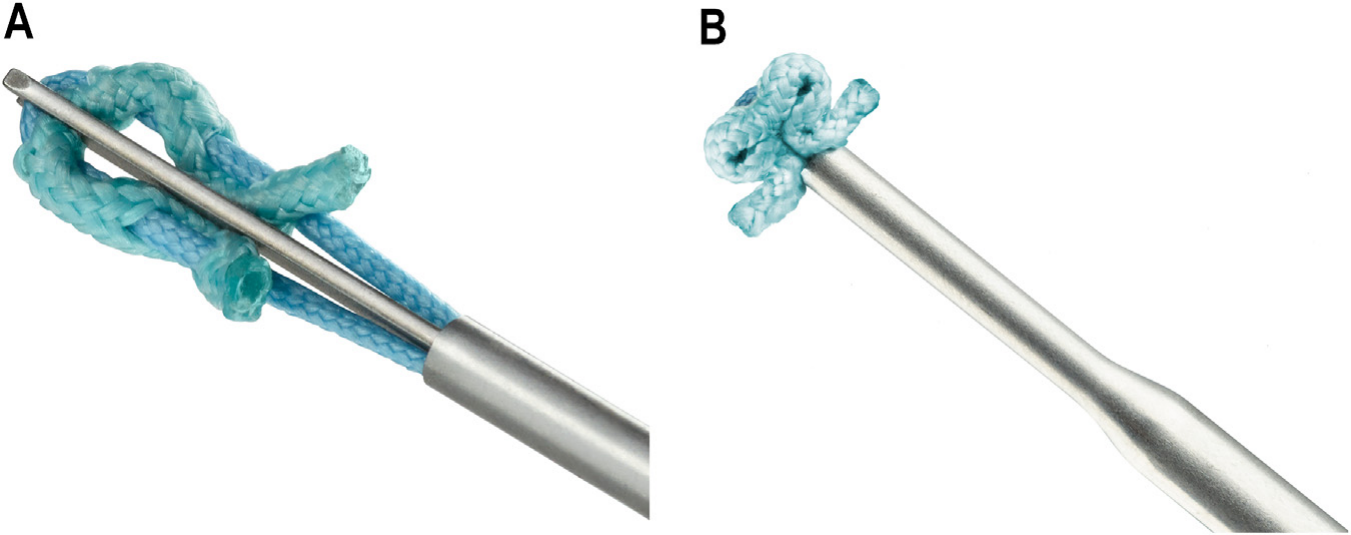
Second-generation all-soft suture anchors used in the present study: an anchor before deployment (A) and after deployment (B).

Patients were enrolled consecutively in accordance with their date of surgery. Patients received second-generation all-soft suture anchors (SUTUREFIX; Smith and Nephew, Memphis, TN, USA Fig. 1 *A* and *B*). These anchors are 1.7 mm and 1.9 mm in size and consist of a soft suture anchor with attached nonabsorbable suture(s) preassembled to either a rigid (SUTUREFIX ULTRA; Smith and Nephew, Memphis, TN, USA) or flexible (SUTUREFIX CURVED; Smith and Nephew, Memphis, TN, USA) insertion device. Anchors are designed in multiple suture configurations, including single-loaded and double-loaded suture options. For double-loaded anchors, the drill hole diameter is 1.9 mm; the depth in the bone is 20.1 mm for SUTUREFIX ULTRA and 19 mm for SUTUREFIX CURVED. A deployed anchor measures 4.4 mm × 4.5 mm × 3.1 mm. These dimensions are slightly smaller for a single-loaded anchor. All surgeons who participated in the study had experience working with these devices. Anchor type was left to the discretion of the surgeon.

All patients underwent postsurgical rehabilitation, which followed the standard practice, according to the surgeon’s preference, at that site.

### Study outcomes

The primary outcome of the study was the clinical success rate, defined as percentage of patients without signs of failure such as recurrent, persistent joint line pain, apprehension, subluxation, dislocation, and/or reintervention at 6 months postoperation, as assessed by the surgeon.

Secondary outcomes were the clinical success rate, defined as percentage of patients without signs of failure and/or reintervention at 12 months postoperation, as assessed by the surgeon; intraoperative anchor deployment success rate; number of anchors pulled out; use of additional anchors, as a result of intraoperative failure; device-related reintervention; and PROs at 6 and 12 months, including pain assessment with visual analog scale (VAS), satisfaction assessment with VAS, EQ-5D-5L Index Score, and EQ-5D-5L VAS Health Score, Rowe Shoulder Score for Instability (ROWE), American Shoulder and Elbow Surgeons (ASES) score, and Constant-Murley Shoulder (CMS) Score. PROs were assessed optionally according to the surgeon’s preference.

Due to the COVID-19 pandemic, in-person visits were instead conducted via telephone. This meant that physical examinations could not take place, and therefore missing data were expected for the ROWE and CMS scores, which were not validated for telephone use. This change affected only the analysis of the PROs but had no impact on the conduct of the study.

Safety was defined as the presence/absence of adverse events (AEs). These were defined as any untoward medical occurrence, disease or injury, or clinical signs. Safety evaluation at all time points was then conducted to assess whether the AEs possibly or definitely related to the study device, or were serious adverse events (SAEs), or serious adverse device effects (SADEs).

### Statistical analysis

All statistical calculations were made using SAS software (SAS Institute, Cary, NC, USA). Resulting *P* values were quoted and 95% 2-sided confidence intervals (CIs) generated where appropriate. Statistical significance was set at *P* < .05.

The present study is exploratory and noncomparative in nature. The sample size selected for this study was precision-based and not based on statistical power considerations. Although biases will be inevitable in this type of study, the use of multiple recruitment centers and well-defined inclusion/exclusion criteria ensures that the study is more representative of a larger population and varying backgrounds. The exploratory data analysis planned for the study, which includes CIs for the outcome summaries, a predefined CI for the primary outcome and exploratory statistical models adjusting for potential confounding factors, is designed to maximize the validity of the study results.

## Results

### Baseline demographics

Forty-one patients were enrolled and underwent treatment (Table I) with 96 sutures being deployed. Although 35 (85.4%) patients finished the study COVID restrictions meant only 32 of these had a formal 12-month call or visit. The 6 remaining patients who did not complete the study were lost to follow-up.

**Table I:** Patient demographics and clinical characteristics.

Variable	Value
Total patients, N	41
Age, y	
Mean ± standard deviation	28.2 ± 8.9
Median	25.0
Sex, N (%)	
Female	5 (12.2%)
Male	36 (87.8%)
Body mass index, kg/m^2^	
Mean ± standard deviation	23.9 ± 3.1
Median	23.7
Surgical indications, N	
Capsular stabilization	33
Bankart repair	36
Anterior shoulder instability	17
SLAP lesion repairs	7
Capsular shift or capsulolabral reconstructions	4
Device, N (%)	
ULTRA	31 (75.6%)
CURVED	16 (39.0%)

*SLAP*, superior labrum anterior and posterior.

### Outcomes

The primary outcome—clinical success at 6 months—was achieved in 27 of the 28 patients (96.4% [95% CI, 81.7%-99.9%]) seen at this time point (Table II). A secondary outcome of clinical success at 12 months was achieved in 31/32 patients (96.9%, [95% CI, 83.8%-99.9%]) seen at this point. Twenty-five patients were available for measurement at both time points (n = 3 [6 months] and n = 7 [12 months] only had measurements at a single time point). No patients experienced an intraoperative suture anchor failure (100% intraoperative anchor deployment success rate) or had a suture anchor that pulled out; hence, there were no additional anchors used and no device-related reinterventions. Intraoperatively, the mean number of sutures successfully deployed per patient was 3.0 (standard deviation, 1.04). The mean number of anchors per patient was 3.0 (standard deviation, 1.04).

**Table II:** Clinical success rate postoperatively by indication.

Procedure	Clinical success at 6 mo postop N = 28				Clinical success at 12 mo postop N = 32			
	Total	Suturefix ultra N = 22	Suturefix curved N = 7	Both suturefix ultra and suturefix curved N = 1	Total	Suturefix ultra N = 25	Suturefix curved N = 13	Both suturefix ultra and suturefix curved N = 6
Capsular stabilization, n (%)∗	20	15 (75)	6 (30)	1 (5)	25	19 (76)	12 (48)	6 (24)
Bankart repair, n (%)∗	24	19 (78.16)	6 (25)	1 (4.17)	28	22 (78.57)	12 (42.86)	6 (21.43)
Anterior shoulder instability, n (%)∗	11	9 (81.81)	3 (27.27)	1 (9.09)	14	11 (78.57)	8 (57.14)	5 (35.71)
SLAP lesion repair, n (%)∗	7	7 (100)	0 (0.00)	0 (0.00)	7	7 (100)	0 (0.00)	0 (0.00)
Capsular shift or capsulolabral reconstruction, n (%)∗	2	1 (50)	1 (50)	0 (0.00)	3	2 (66.67)	2 (22.67)	1 (33.33)
Rotator cuff tear repairs, n (%)∗	0	0 (0.00)	0 (0.00)	0 (0.00)	0	0 (0.00)	0 (0.00)	0 (0.00)
Biceps tenodesis, n (%)∗	0	0 (0.00)	0 (0.00)	0 (0.00)	0	0 (0.00)	0 (0.00)	0 (0.00)
Acromioclavicular separation, n (%)∗	0	0 (0.00)	0 (0.00)	0 (0.00)	0	0 (0.00)	0 (0.00)	0 (0.00)
Deltoid repair, n (%)∗	0	0 (0.00)	0 (0.00)	0 (0.00)	0	0 (0.00)	0 (0.00)	0 (0.00)

Statistically significant improvements over the baseline were reported at 6 and 12 months for most of PROs (Tables III and IV). Change from baseline did not reach statistical significance in CMS Score at 6 months. Change in VAS Satisfaction Score from baseline is not reported as the question relates to satisfaction with the arthroscopic surgery and therefore a preoperative score is not relevant.

**Table III:** Secondary clinical outcomes at baseline and all postoperative follow-up visits (VAS and EQ-5D-5L).

	VAS					
	VAS pain score, 0-10 points			VAS satisfaction score, 0-10 points		
	Baseline (N = 35)	6 mo (N = 28)	12 mo (N = 28)	Baseline N/A†	6 mo (N = 28)	12 mo (N = 28)
Mean (SD)	3.3 (3.1)	1.2 (1.8)	0.4 (1.0)	N/A†	1.6 (2.7)	0.4 (1.1)
*P* value∗	–	.001	<.0001	–	N/A†	N/A†
	EQ-5D-5L					

	EQ-5D-5L index score, 0-1 points			EQ-5D-5L VAS health score, 0-100 points		
Baseline (N = 35)	6 mo (N = 27)	12 mo (N = 29)	Baseline (N = 35)	6 mo (N = 27)	12 mo (N = 29)
Mean (SD)	0.7 (0.2)	0.9 (0.1)	1.0 (0.1)	78.0 (17.7)	87.4 (16.3)	95.1 (7.1)
*P* value∗	–	<.0001	<.0001	–	.03	<.0001

*SD*, standard deviation; *VAS*, visual analog scale.

∗ Change from baseline.

† N/A, Not applicable (The VAS, satisfaction asks about satisfaction with the arthroscopy so is not relevant prior to surgery).

**Table IV:** Secondary clinical outcomes at baseline and all postoperative follow-up visits (ROWE, ASES, CMS).

	ROWE score, 0-100 points			ASES shoulder score, 0-100 points			CMS score, 0-100 points		
	Baseline N = 29	6 mo N = 23	12 mo N = 28	Baseline N = 35	6 mo N = 28	12 mo N = 32	Baseline N = 26	6 mo N = 23	12 mo N = 28
Mean (SD)	38.3 (20.1)	93.3 (11.5)	97.9 (8.5)	66.9 (22.5)	91.0 (12.2)	95.7 (8.0)	69.5 (16.2)	77.9 (6.0)	79.8 (3.6)
*P* value∗	–	<.0001	<.0001	–	<.0001	<.0001	–	.06 (N = 20)†	.002 (N = 25)†

*ASES*, American Shoulder and Elbow Surgeons; *CMS*, Constant-Murley Shoulder; *ROWE*, Rowe Shoulder Score for Instability; *SD*, standard deviation.

∗ Change from baseline.

† The number of patients with the corresponding score at baseline used to calculate the *P* value is N for that time point, except when noted otherwise in parentheses.

### Safety

No AEs were determined to be possibly or definitely related to the study device. One patient (2.4%) experienced a possible SAE and 1 patient (2.4%) a possible SADE. A 23-year-old male experienced traumatic left shoulder dislocation, which was classified as SAE. The patient was not hospitalized and was treated with rest, as well as physiotherapy and medication therapy. The SAE was considered resolved at 1-year follow-up.

The SADE was experienced by a 25-year-old male, who initially underwent capsular stabilization and Bankart repair, and was found to be unrelated to the study device and possibly related to the study procedure. More than 4 months later, the patient experienced recurrent instability and was revised with an open Latarjet procedure. The event was marked as recovering/resolving, and recovery to date has been satisfactory. The patient was not withdrawn from the study.

## Discussion

In this prospective, multicenter study, we demonstrated that the use of second-generation all-soft suture anchors is associated with high rate of clinical success at 6 and 12 months after arthroscopic repair of shoulder labral tears. A statistically significant improvement in several patient-reported measures of shoulder pain, function, and instability was observed at both time points. The anchors demonstrated favorable safety and intraoperative performance, as well as patient satisfaction.

Because second-generation all-soft suture anchors represent a relatively recent design, few studies reporting biomechanical characteristics, clinical outcomes, and safety of these anchors in arthroscopic surgery have been published to date. Erickson et al^7^ compared first-generation and second-generation all-soft sutures in a human cadaveric study and found that load to 2-mm displacement and ultimate load to failure strength was improved in the second-generation (Suturefix Ultra S 1.7 mm) anchors. This is thought to be due to the enhanced locking mechanisms and deployment systems. A systematic review of all-soft suture anchors has also found that they have similar or better biomechanical properties when compared to bioabsorbable, biocomposite, or PEEK anchors.^6^ However, they also recognize that the biomechanical performance is also related to other factors such as bone density and anchor insertion angle. This highlights the importance of studying the clinical outcomes in a range of patients and with multiple surgeons.

Overall, in arthroscopic shoulder repair, the use of second-generation all-soft suture anchors has been associated with favorable outcomes and safety. In a prospective cohort undergoing an arthroscopic Latarjet procedure with Bankart repair, 99% (75/76) of patients had a stable shoulder at the average follow-up of 14 months (range, 6-24 months). The only PRO reported was the mean postoperative ROWE score at the latest follow-up of 95 months (range, 84-100). No recurrent dislocations or hardware failures were reported at that time point.^4^ On longer follow-up, outcomes remain favorable. In a retrospective study, Uzun et al demonstrated a significant improvement from the preoperative levels in the ROWE, CMS, VAS-Pain, and ASES scores at 32 ± 7.4 months in patients undergoing arthroscopic Bankart repair. Recurrent dislocation occurred in 5.6% (4/71) of the study population, and no significant perioperative or postoperative complications were reported.^22^ In a prospective cohort undergoing arthroscopy-assisted massive, irreparable rotator cuff tear, Ozturk et al demonstrated a significant improvement in CMS, VAS, and ASES scores at 31 months. No device-related AEs were reported.^13^ Our findings are consistent with these published reports in terms of recurrent instability, reoperation, SAEs related to the investigational device, and postoperative PROs, confirming short-term safety and clinical effectiveness of these anchors.

First-generation all-soft suture anchors have been studied more extensively. In shoulder labral repair, rotator cuff repair, and Bankart repair, first-generation all-soft suture anchors in the short term have demonstrated clinical success, PROs, and safety comparable with the results of the present study.^2^^,^^23^^,^^8^^,^^9^^,^^11^^,^^19^^,^^21^ The mean postoperative PRO scores ranged in these studies as follows: 0.9-2.1 for VAS-Pain, 78.4-84.3 for CMS, and 92.4-93.7 for ASES at mean follow-up of 13.6-27.8 months. Reoperation incidence ranged from 2.9% (1/35) to 6.1% (2/33) and followed a traumatic recurrent dislocation, in most cases, as opposed to hardware failure. Van der Bracht reported one repeat arthroscopy due to one loose lateral anchor.^23^ Although the second-generation anchors used in this noncomparative study exhibited similar outcomes, they differ in that they are mechanically, rather than manually, tensioned, which could yield improvements in performance.^3^

Our study has several limitations, which should be considered when interpreting its results. First, the single-arm design limits our ability to compare these results against those of first-generation all-suture anchors, which would better allow us to attribute clinical success to improvement in anchor design. Second, the lack of radiologic measures prevents us from assessing the degree of osteolysis or cyst formation and establishing correlation between the observed clinical success and pathology at the anchor site. Anchor site reactions have been reported for all-soft suture anchors and linked to retear after arthroscopic rotator cuff repair.^14^^,^^15^ In 2 recent studies, Ruiz Iban et al found osteolysis associated with the use of all-soft suture anchors to be procedure-related. Both first-generation and second-generation all-soft suture anchors were used in the study. Large cysts at the anchor site were seen in the humeral head after remplissage surgery for shoulder instability but not in the glenoid bone following arthroscopic Bankart repair of shoulder instability at 12 months.^16^^,^^17^ Other reports support these findings.^18^^,^^20^ Hence, anchor-site pathology in the glenoid bone was not expected in the present study. Additionally, patients were monitored over a relatively short follow-up period. However, the 6-month and 12-month time points used in this study align well with published healing and return to activity times following arthroscopic repair of labral tears.^1^^,^^22^^,^^24^ A further limitation to the study is that postoperative range of motion is not reported; however, loss of functional movement is rare after arthroscopic labral repair. Although range of motion is not reported in the results, any problems relating to stiffness would have been identified during the follow-up consultations and recorded as AEs if necessary. A future study, particularly involving radiographic evaluation and longer term follow-up, would help confirm that safety and performance results are maintained in the longer term.

## Conclusion

This study demonstrates favorable performance and safety profiles of the second-generation all-soft suture anchors on short-term follow-up of 6 and 12 months. These outcomes are comparable to those reported for other all-soft suture anchors. The low rate of AEs associated with the device, a high clinical success rate, and significant improvement in the measures of shoulder pain, function, and instability, as well as patient satisfaction, indicate the suitability of these anchors for arthroscopic repair of labral tears in patients with shoulder instability.

## Acknowledgments

The authors acknowledge Anna Brynskikh, PhD, John Watson, and Joseph Mozingo, PhD, for providing medical writing and editing support, and Michelle Foster for providing statistical support for this work. These contributors are employees of Smith + Nephew, Inc., which funded their contributions. The authors also acknowledge Mary Jones, MSc, Fortius Clinic, London, for providing medical writing and editing support for the revised submission.

## Disclaimers

Funding: Funding for this study was provided by 10.13039/100009026Smith + Nephew. These funds supported data collection, data analysis, and the preparation of or editing of the manuscript.

Conflicts of interest: Andrew L. Wallace: Smith + Nephew and Arthrex. Emilio Calvo: Smith + Nephew, DePuy Johnson & Johnson, and Stryker; held paid or unpaid roles in European Society for Surgery of the Shoulder and the Elbow, Journal of Shoulder and Elbow Surgery, and Shoulder Committee of ISAKOS. Jordi Ardèvol Cuesta: Smith + Nephew, SETRADE, and Ospital Asepeyo Sant Cugat; held paid or unpaid roles in Spanish Society of Sport Trauma and SETRADE. Riccardo Lanzetti: Smith + Nephew, Lima Corporate, Zimmer Inc., Arthrex, and Exactech. Gonzalo Luengo-Alonso: Smith + Nephew. Andrew Rokito: Smith + Nephew. Edwin Spencer: Smith + Nephew. Marco Spoliti: Smith + Nephew, Lima Corporate, Zimmer Inc., Exactech, DePuy Johnson & Johnson, and Arthrex.
